# Unicompartmental knee arthroplasty: A PearlDiver study evaluating complications rates, opioid use and utilization in the Medicare population

**DOI:** 10.1186/s40634-021-00390-7

**Published:** 2021-11-09

**Authors:** Brandon L. Morris, Jack M. Ayres, Daniel Reinhardt, Armin Tarakemeh, Scott Mullen, J. Paul Schroeppel, Bryan G. Vopat

**Affiliations:** Department of Orthopedic Surgery, University of Kansas Medical Center, 3901 Rainbow Bvld, Kansas City, KS 66160 USA

**Keywords:** Unicompartmental knee arthroplasty, Total knee arthroplasty, Opioid, Postoperative complication, Medicare, Database

## Abstract

**Purpose:**

Despite increased utilization of unicompartmental knee arthroplasty (UKA) for unicompartmental knee osteoarthritis, outcomes in Medicare patients are not well-reported. The purpose of this study is to analyze practice patterns and outcome differences between UKA and TKA in the Medicare population. It is hypothesized that UKA utilization will have increased over the course of the study period and that UKA will be associated with reduced opioid use and lower complication rates compared to TKA.

**Methods:**

Using PearlDiver, the Humana Claims dataset and the Medicare Standard Analytic File (SAF) were analyzed. Patients who underwent UKA and TKA were identified by CPT codes. Postoperative complications were identified by ICD-9/ICD-10 codes. Opioid use was analyzed by the number of days patients were prescribed opioids postoperatively. Survivorship was defined as conversion to TKA.

**Results:**

In the Humana dataset, 7,808 UKA and 150,680 TKA patients were identified. 8-year survivorship was 87.7% (95% CI [0.861,0.894]). Postoperative opioid use was significantly higher after TKA (186.1 days) compared to UKA (144.7 days) (*p* < 0.01, Δ = 41.1, 95% CI = [30.41, 52.39]). In the SAF dataset, 20,592 UKA patients and 110,562 TKA patients were identified. Survivorship was highest in patients > 80 years old and lowest in patients < 70 years old. In both datasets, postoperative complication rates were higher in TKA patients compared to UKA patients in nearly all categories.

**Conclusions:**

UKA represents an increasingly utilized treatment for osteoarthritis in the Medicare population and may be comparatively advantageous to TKA due to reduced opioid use and complication rates after surgery.

**Level of evidence:**

Level III

## Background

Unicompartmental knee arthroplasty (UKA) represents a surgical treatment option for patients who present with unicompartmental knee osteoarthritis (OA). Medicare beneficiaries undergoing joint arthroplasty procedures has increased as the United States population has aged [7]. Additionally, there is an increasing incidence of patients with unicompartmental knee arthritis that present at an age and activity level less than ideal for a total knee arthroplasty (TKA) [15]. UKA represents a less-invasive alternative to TKA for many of these patients [1, 2]. Coupled with improved instrumentation and understanding of surgical technique, utilization of UKA in recent years has accelerated [10]. What remains unknown is the extent to which patients in the Medicare population are electing to proceed with UKA in lieu of TKA.

Reported advantages of UKA as compared to TKA include bone stock preservation, less surgical exposure and shorter operating time, improved post-operative knee motion and kinematics, lower blood loss and transfusion rates, lower infection rates, shorter inpatient stay, accelerated rehabilitation, and lower implant costs [1]. Indications for UKA include lower activity demand patients, functional and painless knee range of motion, preserved joint alignment and stability, correctible axial malalignment and lack of significant patellofemoral or contra-compartmental osteoarthritis [2, 13].

Survivorship studies have demonstrated promising UKA survival data with predictable outcomes in patients undergoing conversion to total knee arthroplasty: a 2010 literature review reports 10-year survivorship at single-center studies to be between 95 and 98% and 15-year survivorship between 85 and 96% [6]. Despite these advantages, UKA utilization and survivability remains unknown within the Medicare population. The purpose of this study is to analyze UKA practice patterns in the Medicare population and analyze implant survival, opioid use and postoperative complication rates between UKA and TKA. It is hypothesized that UKA utilization will have increased over the course of the study period and that UKA will be associated with reduced opioid use and lower complication rates compared to TKA.

## Methods

In this study, two datasets were examined utilizing the PearlDiver Application. The PearlDiver database is a publicly available, Health Insurance Portability and Accountability Act (HIPAA)-compliant national database containing Current Procedural Terminology (CPT), International Classification of Diseases, Ninth Revision (ICD-9), and International Classification of Diseases, Tenth Revision (ICD-10) codes related to orthopedic procedures. Institutional review board approval was not required for the study. The first dataset is the Humana Claims dataset which contains all medical, pharmaceutical and lab claims from 24.27 million Humana patients from 2007 through March 2017. The Humana dataset contains commercially-insured as well as Medicare patients, therefore search queries were filtered such that we analyzed patients with Medicare Advantage plans through Humana. The second dataset is the Medicare Standard Analytic File (SAF) which contains information of 51 million Medicare patients from 2005 to 2014 based on inpatient and outpatient facility billing records. Since Medicare Advantage patients in the Humana dataset are also listed in the SAF dataset, results are reported by dataset to optimize data accuracy.

UKA patients were identified by CPT-27446, which captures medial or lateral compartment UKAs. TKA patients were identified by CPT-27445 and CPT-27447. Knee arthroplasty revisions were identified by CPT-27486 and CPT-27487. Year of surgery, demographic data, complications as reported by ICD-9 and ICD-10 coding (Appendix C), and comorbidity index scores (Appendix A) were extracted from the datasets. Demographic data included patient age at the time of surgery, gender and region. Regions were defined by Midwest: IA, KS, MN, MO, NE, IL, IN, MI, WI, OH, ND, SD; Northeast: CT, MA, ME, NH, NJ, PA, RI, NY, VT; South: AL, AR, DC, DE, FL, GA, KY, LA, MD, MS, NC, OK, SC, TN, TX, VA, WV, PR; and West: AK, AZ, CA, CO, ID, MT, NM, NV, OR, UT, WA, WY, HI.

Elixhauser and Charlson comorbidity measures are well-known risk adjustment models commonly used for “adjustment of quality and safety data” [14]. These scores were calculated for each patient and reported in aggregate for each group in each database in order to compare the relative health of each population. The comorbidities included in each calculation and methodology is explained in Appendix A.

Opioid use was calculated by identifying filled postoperative opioid prescriptions (listed in Appendix B). Because pharmaceutical information is not available in the SAF dataset, opioid use analysis was not performed on that data set.

Statistical analysis was performed using qualitative analysis and logistic regression. P values of < 0.05 were considered significant. Statistical analysis on opioid use and time between UKA and TKA/revision were performed with use of the Pearl Diver Bellwether application. For statistical analysis using proportions of UKA/TKA patients experiencing an event (such as complication or revision), Chi-square tests were conducted using R[v 3.6.3]. Elixhauser and Charlson scores were analyzed using Mann–Whitney U tests in R[v 3.6.3]. For the time between UKA and TKA and the time until revision, z-tests were used and difference with regards to age ranges were analyzed using ANOVA in R[v 3.6.3]. Survivability was defined as conversion to TKA and Kaplan–Meier survival curves were generated by the PearlDiver Bellwether application. Kaplan–Meier survival curves were then compared using a log rank test which approximates a Χ^2^ test statistic for which X^2^ values greater than the reference value of 3.84 are considered significant.

## Humana results

### UKA utilization and demographics

**Fig. 1:**
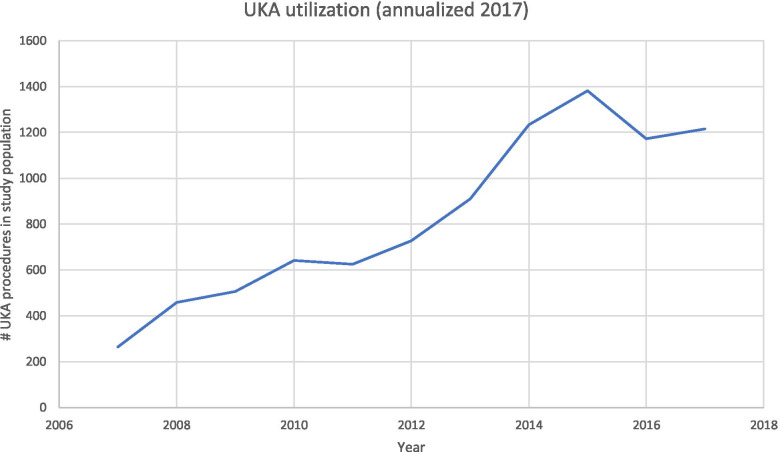
UKA utilization by year

Between 2007 and March 2017, 7,808 patients underwent UKA in the Medicare Advantage population (Table 1). 150,680 patients underwent TKA in the same time period. Annual utilization trends varied during the study period (Table 2). Though TKA was performed more commonly in every year as compared to UKA, UKA utilization generally increased over time from 264 cases in 2007 to 1,382 in 2015. During this time period, the percentage of UKA cases compared to UKA and TKA cases combined rose from 3.79% to 4.96% (Table 2; Fig. 1).

**Table 1:** All patients who underwent UKA and TKA during the study period

	**UKA**		**TKA**	
**Total**	7,808		150,680	
	#	%	#	%
**Age**				
64 and under	977	13%	21,325	14%
65–69	2,208	28%	40,708	27%
70–74	2,303	29%	42,825	28%
75–79	1,427	18%	30,510	20%
80–84	723	9%	15,273	10%
85 and older	304	4%	6,104	4%
**Region**				
Midwest	2,198	28%	43,775	29%
Northeast	183	2%	4,167	3%
South	4,678	60%	87,067	58%
West	750	10%	15,718	10%
**Gender**				
Female	4,216	54%	96,536	64%
Male	3,592	46%	54,144	36%
**Race**				
White	6,547	84%	119,440	79%
Black	345	4%	14,487	10%
Asian	34	0%	656	0%
Hispanic	53	1%	1,565	1%
Native American	14	0%	341	0%
Other	75	1%	1,522	1%
Unknown	740	9%	12,669	8%

**Table 2:** Number of patients undergoing UKA or TKA by year

	UKA	TKA	TKA:UKA	%UKA of cases
2007	264	6709	25.41	3.79%
2008	458	8793	19.20	4.95%
2009	506	10,405	20.56	4.64%
2010	641	12,960	20.22	4.71%
2011	625	14,637	23.42	4.10%
2012	728	15,976	21.95	4.36%
2013	911	19,697	21.62	4.42%
2014	1233	22,793	18.49	5.13%
2015	1382	26,480	19.16	4.96%
2016	1172	22,603	19.29	4.93%
Annualized 2017	1216	23,400	19.24	4.94%

Patients aged 70–74 years accounted for the most UKA procedures (2,303), followed by patients aged 65–69 years (2,208) (Table 1). Gender utilization was 54% female and 46% male. Geographically, most procedures were performed in the South (60%). Age profile and geographic predominance were similar to the TKA group; however, 64% of patients who underwent TKA were women.

The median Elixhauser adjustment for TKA is 7 compared to 6 for UKA (*p* < 0.01). There was a statistically significant difference in Charlson scores (*p* < 0.01) (Table 3).

**Table 3:** Elixhauser and Charlson comorbidity adjustment scores for UKA and TKA

Adjustment score	Elixhauser		Charlson	
	**UKA**	**TKA**	**UKA**	**TKA**
	# of patients with the Elixhauser/Charlson score			
0	101	968	2,753	44,544
1	303	3,636	1,760	34,437
2	578	7,145	1,200	23,837
3	733	10,288	799	17,062
4	762	12,711	470	10,540
5	800	13,670	285	7,207
6	732	13,719	205	4,723
7	686	13,492	106	2,930
8	577	12,395	106	2,052
9	501	11,257	47	1,271
10	450	10,012	34	792
11	389	8,832	17	489
12	294	7,509	11	291
13	252	6,411	-	184
14	190	5,198	-	125
15	153	4,135	-	88
16	99	3,019	-	52
17	90	2,236	-	24
18	51	1,581	-	-
19	23	1,084	-	-
20	22	651	-	-
21	11	385	-	-
22	-	191	-	-
23	-	106	-	-
24	-	35	-	-
25	-	11	-	-
	Overall			
Average	7.04	8.06	1.79	2.07
Median	6.00	7.00	1.00	1.00
St. dev	4.18	4.35	2.17	2.33
***p***	** < 0.0001**		** < 0.0001**	

### UKA conversion and revision

Table 4 shows data from the Humana dataset related to UKA conversion to TKA. 358 UKA (4.59%) patients underwent conversion to TKA with an average time between UKA and TKA of 817 days (Standard deviation (SD) = 699.070). Female patients were not significantly more likely to undergo conversion to TKA at 5.00% compared to 4.09% of men (*p* = 0.055, RR = 0.818, 95% CI = [0.667, 1.005), however, time between UKA and TKA conversion was significantly longer for female patients (852 days) compared to male patients (767 days) (*p* < 0.001, Δ = 85.1, 95% CI = [53.5, 119.8]). Conversion to total knee arthroplasty occurred more commonly in patients under the age of 70 with 6.07% of patients undergoing conversion compared to 4.06% of patients ages 70–79 (*p* < 0.01, RR = 0.67, 95% CI = [0.54, 0.82]) and 1.57% of patients over 80 (*p* < 0.01, RR = 0.26, 95% CI = [0.16, 0.43]). Regarding time between UKA and reversion to TKA, the ANOVA test for differences in age showed a significant result (*p* < 0.001). Patients under 70 had longer times between UKA and conversion to TKA than patients aged 70–79 (Δ = 75.8, 95% CI = [44.1, 107.5]) and patients over 80 (Δ = 171.2, 95% CI = [124.2, 218.3]). Patients 70–79 also had longer times between UKA and conversion to TKA than patients over 80 (Δ = 95.4, 95% CI = [49.02, 141.7]).

**Table 4:** Time between UKA and TKA for patients undergoing conversion

	Number of UKA patients	UKA patients who underwent conversion to TKA					Average time between UKA and TKA for patients undergoing conversion		
	#	#	%	p	RR	95% CI	Days	Δ	95% CI
**Total**	7,808	358	4.59%	-	-	-	816.997 (SD 699.070)	-	-
**Age at UKA**									
< 70	3,180	193	6.07%	-	-	-	857.802 (SD 757.430)	-	-
70–79	3,694	150	4.06%	< 0.0001	0.6691	(0.5434, 0.8238)	782.020 (SD 641.146)	75.802	(44.144, 107.460)
> 80	1,021	16	1.57%	< 0.0001	0.2582	(0.1558, 0.4278)	686.563 (SD 414.917)	171.239	(124.164, 218.314)
**Gender**									
Female	4,216	211	5.00%	-	-	-	851.957 (SD 659.386)	-	-
Male	3,592	147	4.09%	0.0547	0.8177	(0.6656, 1.0048)	766.816 (SD 751.853)	85.141	(53.5070, 119.7749)

**Fig. 2:**
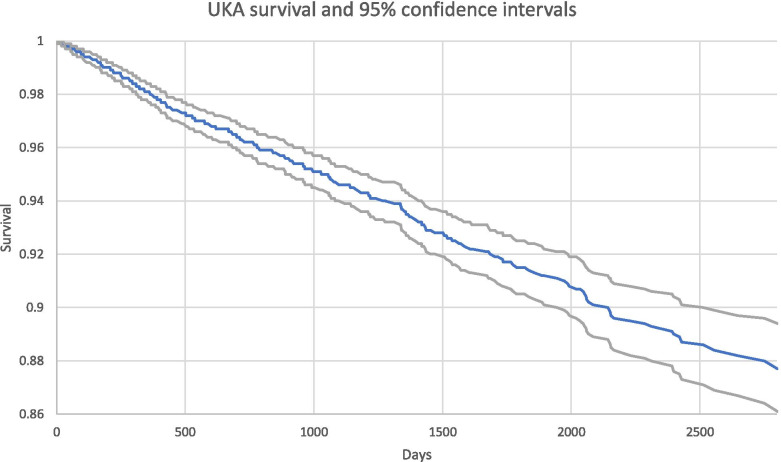
UKA survival and 95% confidence intervals

Overall 8-year UKA implant survival was 87.7% (95% CI [0.861,0.894]) (Fig. 2).

Table 5 shows data from the Humana dataset related to revision surgeries for both TKA and UKA. Revision was more common after UKA than TKA as 303 UKA patients (3.88%) underwent a revision and 3,921 TKA patients (2.60%) underwent a revision (*p* < 0.01, RR = 1.49, 95% CI = [1.33, 1.67]). Additionally, revision occurred later following UKA compared to TKA in patients who underwent revision. Average time between TKA and revision was 608 days (SD = 603.241) days, whereas average time between UKA and revision was 718 days (SD = 691.332) (*p* < 0.001, Δ = 110.7, 95% CI = [95.1, 126.3]). Out of the 7,808 UKA patients, 358 were converted to TKA, 303 were revised, and 49 were both converted and revised.

**Table 5:** Time until revision for UKA and TKA

	**UKA Patients**	UKA revisions					Avg. time between UKA and revision		
		#	%	p	RR	95% CI	Mean (SD)	Δ	95% CI
**Total**	7,808	303	3.88%	-	-	-	718.535 (SD 691.332)	-	-
**Age at UKA**									
< 70	3,180	166	5.22%	-	-	-	715.880 (SD 653.431)	-	-
70–79	3,694	111	3.00%	< 0.0001	0.5756	(0.4548, 0.7285)	742.694 (SD 755.353)	-26.814	(-59.798, 6.170)
> 80	1,021	28	2.74%	< 0.0001	0.5254	(0.3542, 0.7792)	656.179 (SD 589.094)	59.7	(10.7, 108.7)
**Gender**									
Female	4,216	173	4.10%	-	-	-	726.509 (SD 677.448)	-	-
Male	3,592	130	3.62%	0.2695	0.882	(0.7056, 1.01024)	708.923 (SD 711.889)	17.586	(-13.4002, 48.5722)
	**TKA patients**	TKA revisions					Avg. time between TKA and revision		
		#	%	p	RR	95% CI	Mean (SD)	Δ	95% CI
**Total**	150,680	3,921	2.60%	-	-	-	607.849 (SD 603.241)	-	-
**Age at TKA**									
< 70	61,030	2,177	3.57%	-	-	-	647.463 (SD 620.240)	-	-
70–79	71,702	1,510	2.11%	< 0.0001	0.5904	(0.5534, 0.6297)	604.472 (SD 613.365)	42.991	(36.345, 49.637)
> 80	21,081	364	1.73%	< 0.0001	0.4841	(0.4337, 0.5403)	555.085 (SD 610.039)	92.378	(82.738, 102.018)
**Gender**									
Female	96,536	2,448	2.54%	-	-	-	632.112 (SD 626.075)	-	-
Male	54,144	1,473	2.72%	0.3007	1.0728	(1.0066, 1.1435)	576.525 (SD 561.138)	55.587	(49.4276, 61.7464)

In the UKA group, no significant difference was found between male and female patients regarding revision rate (*p* = 0.270 RR = 0.882, 95% CI = [0.706, 1.012]) or time between UKA and revision (*p* = 0.133, Δ = 17.6, 95% CI = [-13.4, 48.6]) (Table 5). Regarding age for UKA patients, patients under 70 years old had no significant difference in times between UKA and conversion to TKA from those ages 70–79 (Δ = -26.8, 95% CI = [-59.8, 6.2]), but longer times between UKA and conversion to TKA than those over 80 years (Δ = 59.7, 95% CI = [10.7, 108.7]). Patients ages 70–79 also had longer times between UKA and conversion to TKA than those over 80 years old (Δ = 86.5, 95% CI = [38.3, 134.7]).

In the TKA group, the revision rate was not significantly different between male and female patients (*p* = 0.301, RR = 1.073, 95% CI = [1.001, 1.144]), however, the time between TKA and revision was longer in female patients compared to male patients (*p* < 0.001, Δ = 55.6, 95% CI = [49.4, 61.7]). The TKA group had significant differences with regards to all age groups. Patients under 70 years old had longer times between UKA and conversion to TKA than those ages 70–79 (Δ = 43.0, 95% CI = [36.3, 49.6]) and over 80 years old (Δ = 92.4, 95% CI = [82.7, 102.0]), while patients ages 70–79 also had longer times between UKA and conversion to TKA than those over 80 years old (Δ = 49.4, 95% CI = [39.9, 58.8]).

### Opioid use

Of the 7,808 patients who underwent UKA, 5,605 patients (71.79%) filled prescriptions for opioids for an average of 144.7 days after surgery (Table 6). Of the 150,680 patients who underwent TKA, 114,583 patients (76.04%) filled prescriptions for an average of 186.1 days after surgery (*p* < 0.001, Δ = 41.1, 95% CI = [52.4, 30.4]) (Table 6).

**Table 6:** Days of opioid use following TKA and UKA

	**UKA**	**TKA**
**Number of patients**	5,605 (71.79% of pts who underwent UKA)	114,583 (76.04% of pts who underwent TKA)
**Number of days filled:**		
Mean	144.7	186.1
Standard deviation	408.02	449.63
*p*-value	< 0.0001	
95% CI	(52.3944, 30.4056)	

### Complications

Complications occurred at a significantly higher rate in all categories except capsulitis (*p* = 0.266) in the TKA group compared to the UKA group (Table 7). This includes cardiovascular complications such as DVT (*p* = 0.022) and cardiac arrest (*p* = 0.008), as well as wound dehiscence (*p* < 0.001) (Table 7).

**Table 7:** Incidence of complications within 12 months following TKA and UKA

Complication	UKA		TKA		*p*	RR	95% CI
	#	%	#	%			
Acute kidney injury	248	3.18%	9,078	6.02%	< 0.0001	0.5581	(0.4930, 0.6318)
Cardiac arrest	25	0.32%	823	0.55%	0.0082	0.5893	(0.3961, 0.8767)
Deep vein thrombosis	42	0.54%	1,168	0.78%	0.0215	0.6991	(0.5143, 0.9503)
Pneumonia	280	3.59%	7,891	5.24%	< 0.0001	0.7194	(0.6399, 0.8086)
Pulmonary embolism	99	1.27%	3,012	2.00%	< 0.0001	0.6466	(0.5299, 0.7888)
Urinary tract infection	1,109	14.20%	28,445	18.88%	< 0.0001	0.8889	(0.8409, 0.9397)
Wound deshiscence	82	1.05%	2,407	1.60%	0.0003	0.6676	(0.5363, 0.8310)
Hematoma	71	0.91%	2,245	1.49%	0.0001	0.6191	(0.4894, 0.7833)
Transfusion	82	1.05%	4,271	2.83%	< 0.0001	0.3808	(0.3064, 0.4733)
Capsulitis	53	0.68%	1,204	0.80%	0.2655	0.8560	(0.6507, 1.1260)
Nerve injury	0	0.00%	81	0.05%	0.0405	0	-

## Medicare Standard Analytic File results

### UKA utilization and demographics

**Fig. 3:**
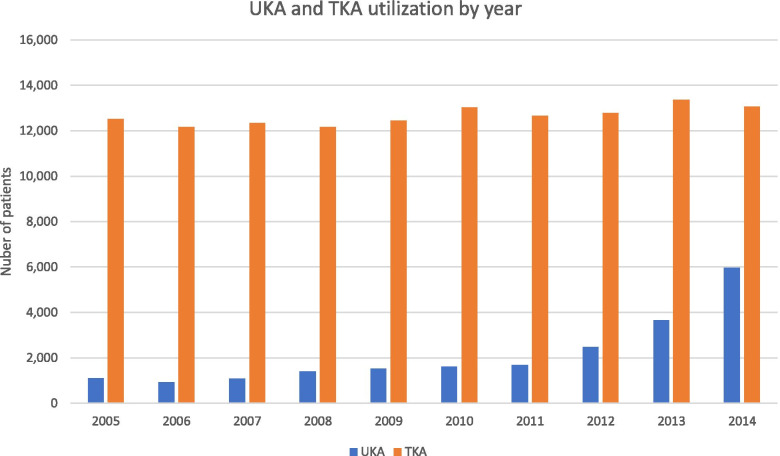
UKA and TKA utilization by year

Between 2005 and 2014, a total of 20,592 patients underwent UKA procedures (Table 8). 110,562 patients underwent TKA in the same time period. Annual utilization trends varied during the study period with the most dramatic increase in UKA utilization occurring in years 2012–2014 (Fig. 3). TKA was performed more commonly in every year compared to UKA, although TKA did not show as large of a rate of increase as UKA during the last three years of the study period. UKA made up an increasing proportion of overall knee arthroplasty volume toward the end of the study period, as nearly 1/3rd of knee arthroplasty procedures performed in 2014 were UKA (Fig. 3).

**Table 8:** All patients who underwent UKA and TKA during the study period

	**UKA**		**TKA**	
**Total**	20,592		110,562	
	#	%	#	%
**Age**				
64 and under	1,709	8%	11,183	10%
65–69	6,319	31%	30,628	28%
70–74	5,518	27%	29,247	26%
75–79	4,005	19%	24,685	22%
80–84	2,250	11%	14,539	13%
85 and older	959	5%	5,534	5%
Unknown	147	1%	1,681	2%
**Region**				
Midwest	4,502	22%	30,816	28%
Northeast	2,736	13%	17,703	16%
South	10,627	52%	43,475	39%
West	2,732	13%	18,777	17%
Unknown	0	0%	27	0%
**Gender**				
Female	10,543	51%	71,394	65%
Male	9,933	48%	38,214	35%
Unknown/Other	147	1%	1,681	2%

The largest portion of patients undergoing UKA were aged 65–69 years (6,319), followed by patients aged 70–74 years (5,518) (Table 8). Gender utilization was 51% female and 48% male. Geographically, most procedures were performed in the South (52%). Age group and geographic predominance were similar to the TKA group; however, 65% of patients who underwent TKA were women (35% men).

The median Elixhauser adjustment for TKA is 9 compared to 5 for UKA (*p* < 0.01). The median Charlson adjustment for TKA is 2 compared to 1 for UKA (*p* < 0.01) (Table 9).

**Table 9:** Elixhauser and Charlson comorbidity adjustment scores for UKA and TKA

Adjustment score	Elixhauser		Charlson	
	**UKA**	**TKA**	**UKA**	**TKA**
	Number of patients with the Elixhauser/Charlson score			
0	729	504	8,358	25,981
1	1,749	1,861	5,177	24,946
2	2,418	3,580	3,071	19,094
3	2,493	5,510	1,698	14,143
4	2,287	6,914	893	8,922
5	2,017	8,016	516	5,860
6	1,673	8,509	301	3,663
7	1,452	8,747	190	2,381
8	1,222	8,624	138	1,723
9	935	8,415	87	1,267
10	842	7,992	69	904
11	636	7,470	42	611
12	533	6,589	22	353
13	422	5,946	12	268
14	348	5,172	-	169
15	262	4,259	-	126
16	185	3,440	-	56
17	139	2,904	-	37
18	100	2,197	-	27
19	74	1,565	-	12
20	26	1,007	-	-
21	24	643	-	-
22	15	356	-	-
23	-	199	-	-
24	-	84	-	-
25	-	37	-	-
26	-	16	-	-
	Overall			
Average	5.73	9.30	1.43	2.39
Median	5.00	9.00	1.00	2.00
St. dev	4.11	4.71	1.91	2.54
***p***	** < 0.0001**		** < 0.0001**	

### UKA conversion and revision

Table 10 shows data from the SAF dataset related to UKA conversion to TKA. 447 (2.17%) UKA patients underwent subsequent TKA and the average time between UKA and TKA was 987 days (SD = 764.815) (2.71 years). Male patients were less likely to undergo conversion to TKA at 1.70% compared to 2.50% of women (*p* < 0.01, RR = 0.68, 95% CI = [0.56, 0.82]). However, the time between UKA and conversion was not statistically different between female and male patients (*p* = 0.287, Δ = 6.02, 95% CI = [-14.98, 27.03]). Conversion occurred more commonly in patients under the age of 70 with 2.78% of patients undergoing conversion compared to 1.78% of patients ages 70–79 (*p* < 0.01, RR = 0.64, 95% CI = [0.53, 0.78]) and 1.35% of patients over 80 years old (*p* < 0.01, RR = 0.48, 95% CI = [0.35, 0.67]) (Table 10). Analysis of time between UKA and TKA for age showed no significant difference between patient under 70 years old and patients ages 70–79 (Δ = 8.6, 95% CI = [-13.9, 31.1]). However, patients over 80 years old had shorter times between UKA and conversion to TKA than both patients under 70 years old (Δ = 182.3, 95% CI = [151.3, 213.3]) and patients ages 70–79 (Δ = 173.7, 95% CI = [143.4, 204.1]).

**Table 10:** Time betwen UKA and TKA for patients undergoing conversion

	Number of UKA patients	UKA patients converted to TKA					Average time between UKA and TKA		
		#	%	*p*	RR	95% CI	Days	Δ	95% CI
**Total**	20,592	447	2.17%	-	-	-	987.378 (SD 764.815)	-	-
**Age at UKA**									
< 70	8,018	223	2.78%	-	-	-	988.852 (SD 781.501)	-	-
70–79	9,433	168	1.78%	< 0.0001	0.6404	(0.5253, 0.7806)	980.262 (SD 729.328)	8.590	(-13.921, 31.101)
> 80	3,191	43	1.35%	< 0.0001	0.4845	(0.3505, 0.6698)	806.558 (SD 768.786)	182.294	(151.274, 213.314)
**Gender**									
Female	10,543	264	2.50%	-	-	-	971.417 (SD 746.139)	-	-
Male	9,933	169	1.70%	< 0.0001	0.6795	(0.5613, 0.8226)	965.391 (SD 785.201)	6.026	(-14.9808, 27.0328)

**Fig. 4:**
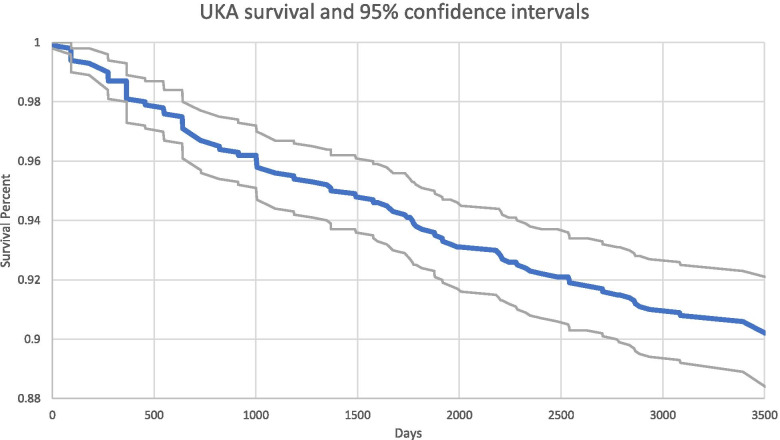
UKA survival and 95% confidence intervals (with a minimum 8 year follow up)

UKA implant survival was 91.1% (95% CI [0.895,0.928]) (Fig. 4; Table 11) at 8 years. Implant survival increased with advancing age (X^2^ = 4.66). The highest rate of survival was seen in patients over 80-years-old (95.6% (95% CI [0.926,0.992])) and the lowest in patients under 70-years-old (89.3% (95% CI [0.865,0.921])) (Table 11).

**Table 11:** 8 year UKA implant survival by age and gender (with a minumum 8 year follow up)

	Number of UKA patients	Convervsion to TKA	8 year survival (95% CI)
**Total**	1195	110	.911 (.895-.928)
**Age**			
< 70	466	54	.893 (.865-.921)
70–79	585	49	.915 (.892-.937)
> 80	144	7	.958 (.926-.992)
**Gender**			
Female	673	70	.899 (.876-.922)
Male	522	40	.927 (.905-.950)

Table 12 shows data from the SAF dataset related to revision surgeries for both TKA and UKA. Revision was less common after UKA than TKA as 364 UKA patients (1.77%) underwent a revision and 3,630 TKA patients (3.28%) underwent a revision (*p* < 0.001, RR = 0.53, 95% CI = [0.48, 0.59]). The percentage of patients who underwent a revision is higher in the UKA group than it is in the TKA group, among all age classes and genders. The difference in time to revision between UKA and TKA patients who underwent revision was statistically significant (*p* < 0.001, Δ = 66.3, 95% CI = [54.9, 77.7]). The overall average time between TKA and revision was 867 (SD = 754) and the overall average time between UKA and revision was 933 (SD = 768) days (Table 12). Of the 20,592 UKA patients, 447 were converted to TKA, 364 were revised, and 67 were both converted and revised.

**Table 12:** Time until revision for UKA and TKA

	**UKA Patients**	UKA revisions					Avg. time between UKA and revision		
		#	%	p	RR	95% CI	mean (SD)	Δ	95% CI
**Total**	20,592	364	1.77%	-	-	-	932.942 (SD 768.273)	-	-
**Age at UKA**									
< 70	8,018	185	2.31%	-	-	-	862.746 (SD 755.779)	-	-
70–79	9,433	140	1.48%	< 0.0001	0.6432	(0.5175, 0.7995)	1039.486 (SD 778.513)	-176.74	(-199.296, -154.184)
> 80	3,191	36	1.13%	< 0.0001	0.4890	(0.3430, 0.6971)	789.528 (SD 697.079)	73.218	(42.137, 104.299)
**Gender**									
Female	10,543	224	2.12%	-	-	-	981.920 (SD 773.567)	-	-
Male	9,933	135	1.36%	< 0.0001	0.6397	(0.5176, 0.7906)	823.778 (SD 739.283)	158.142	(137.420, 178.864)
	**TKA patients**	TKA revisions					Avg. time between TKA and revision		
		#	%	p	RR	95% CI	mean (SD)	Δ	95% CI
**Total**	110,562	3,630	3.28%	-	-	-	866.597 (SD 754.557)	-	-
**Age at TKA**									
< 70	41,518	1,865	4.49%	-	-	-	898.958 (SD 775.442)	-	-
70–79	52,129	1,525	2.93%	< 0.0001	0.6513	(0.6094, 0.6960)	908.423 (SD 772.142)	-9.4650	(-19.220, 0.290)
> 80	19,632	368	1.87%	< 0.0001	0.4173	(0.3736, 0.4660)	718.584 (SD 669.883)	180.3740	(167.529, 193.219)
**Gender**									
Female	71,394	2,279	3.19%	-	-	-	896.808 (SD 761.672)	-	-
Male	38,214	1,211	3.17%	0.8352	0.9927	(0.9269, 1.0632)	814.272 (SD 743.505)	82.5360	(73.2201, 91.8519)

In the UKA group, the revision rate for male patients was lower than female patients (*p* < 0.01, RR = 0.64, 95% CI = [0.52, 0.79]), and revision occurred sooner for male patients compared to female patients (*p* < 0.001, Δ = 158.1, 95% CI = [137.4, 178.9]). UKA patients under 70 years old had less time between UKA and revision than those ages 70–79 (Δ = -176.7, 95% CI = [-199.3, -154.2]), but more time between UKA and revision than those over 80 years old (Δ = 73.2, 95% CI = [42.1, 104.3]). Patients ages 70–79 had more time between UKA and revision than patients over 80 years old (Δ = 250.0, 95% CI = [219.5, 280.4]).

In the TKA group, there was no difference between male and female revision rate (*p* = 0.835, RR = 0.99, 95% CI = [0.93, 1.06]), but the time between TKA and revision was longer in female patients compared to male patients (*p* < 0.001, Δ = 82.5, 95% CI = [73.2, 91.9]). Times between TKA and revision for patients under 70 years old were not significantly different from those ages 70–79 (Δ = -9.5, 95% CI = [-19.2, 0.3]), however, patients under 70 years old had longer times between TKA and revision than patients over 80 years old (Δ180.4, 95% CI = [167.5, 193.2]). Patients ages 70–79 also had more time between TKA and revision than patients over 80 years old (Δ = 189.8, 95% CI = [177.4, 193.2]).

### Complications

Complications occurred at a higher rate in all categories in the TKA group compared to the UKA group (Table 13). Differences in complication rates between TKA and UKA demonstrated statistical significance (*p* < 0.01) (Table 13).

**Table 13:** Incidence of complications within 12 months following TKA and UKA

Complication	UKA		TKA		*p*-value	RR	95% CI
	#	%	#	%			
Acute kidney injury	417	2.03%	5,110	4.62%	< 0.0001	0.4567	(0.4138, 0.5041)
Cardiac arrest	47	0.23%	617	0.56%	< 0.0001	0.4111	(0.3057, 0.5528)
Deep vein thrombosis	13	0.06%	207	0.19%	< 0.0001	0.3378	(0.1929, 0.5915)
Pneumonia	449	2.18%	5,984	5.41%	< 0.0001	0.423	(0.3848, 0.4651)
Pulmonary embolism	192	0.93%	2,704	2.45%	< 0.0001	0.3899	(0.3371, 0.4510)
Urinary tract infection	1,742	8.46%	22,678	20.51%	< 0.0001	0.4905	(0.4683, 0.5139)
Wound dishiscence	140	0.68%	1,327	1.20%	< 0.0001	0.5725	(0.4813, 0.6810)
Hematoma	171	0.83%	1,690	1.53%	< 0.0001	0.5507	(0.4709, 0.6441)
Transfusion	312	1.52%	4,869	4.40%	< 0.0001	0.3582	(0.3198, 0.4013)
Capsulitis	106	0.51%	1,381	1.25%	< 0.0001	0.4169	(0.3423, 0.5076)
Nerve injury	0	0.00%	89	0.08%	< 0.0001	0	-

## Discussion

Unicompartmental knee arthroplasty demonstrated increased utilization between 2005 and 2017 in the Medicare population. Though TKA was performed more commonly in every year as compared to UKA, UKA utilization generally increased over time: In the Humana dataset the UKA cases increased from 264 cases in 2007 to 1,382 in 2015 (Table 2; Fig. 1). In the SAF dataset, nearly 1/3rd of knee arthroplasty procedures performed in 2014 were UKA (Fig. 3). Analysis of opioid use in the Humana dataset revealed a statistically significant (*p* < 0.01) decrease in the mean number of days for which opioid prescriptions were filled after UKA (145 days) as compared to TKA (186 days). UKA was associated with significantly lower complication rates compared to TKA, including complications such as DVT (Humana: (*p* = 0.0215, RR = 0.70, 95% CI = [0.51, 0.95]) and SAF: (*p* < 0.0001, RR = 0.34, 95% CI = [0.19, 0.59])) and cardiac arrest (Humana: (*p* = 0.0082, RR = 0.59, 95% CI = [0.40, 0.88]) and SAF: (*p* < 0.0001, RR = 0.41, 95% CI = [0.31, 0.55])). With the exception of capsulitis in the Humana dataset, all analyzed complications showed a statistically significant reduction (*p* < 0.05) following UKA compared to TKA (Tables 7 and 13). UKA implant survival was 87.7% (95% CI [0.861,0.894]) (Fig. 2) in the Humana dataset and 91.1% (95% CI [0.895,0.928]) (Fig. 4; Table 11) at 8 years in the SAF dataset.

Post-operative opioid use was found to be less in patients undergoing UKA compared to those who underwent TKA. The queried databases provided prescribed days of opioids, which was used as a proxy for opioid use. Although we report higher MME in TKA patients compared to UKA patients, we were not able to provide statistical analysis for that finding. Opioids are used for postoperative pain control and then gradually tapered over the postoperative period. Many patients take opioids during outpatient physical therapy. Although the amount of opioid use may be significantly reduced during the physical therapy window compared to the days/weeks immediately following surgery, prescriptions for opioids would still be filled during the months after surgery when patients are undergoing physical therapy. Additionally, with a median length of 24 days for UKA and 34 days for TKA, the distribution is positively skewed. The maximum number of days of opioid prescriptions was 6,327 days for UKA and 8,191 days for TKA, which suggests that there may be a number of individuals in the study with long-term opioid prescriptions that raise the mean number of days of use. This is especially probable considering the reported 3^rd^ quartile of 48 days for UKA and 110 days for TKA. Fewer patients initiated opioid use postoperatively after UKA compared to TKA. Our results mirror those reported by Burn et al. that UKA was associated with a reduced risk of postoperative opioid use of 0.81, (95% [CI 0.73–0.90]) [4]. Additionally, a 2020 retrospective analysis evaluating patients who underwent UKA between 2015 and 2018 reported the opioid dosage was lower at 160.5 ± 29.3 mg for UKA and 186.1 ± 46.8 mg for TKA (t-2.969, *p* < 0.01) [17].With increasing focus on decreasing opioid use, the usage profile for UKA may prove beneficial [18].

Another key finding of this study was that postoperative complications within the year following UKA procedures were significantly lower (*p* < 0.05) than those following TKA. This trend held true for almost all analyzed complications in both datasets. On average, TKA patients demonstrated poorer baseline health characteristics as measured by Elixhauser and Charlson comorbidity indexes. This is especially true for the SAF dataset which the median Elixhauser adjustment for TKA is 9 compared to 5 for UKA (*p* < 0.01). Our findings may be in part due to differences in baseline health, but we were not able to adjust for these differences in our statistical analysis. However, our findings are similar to other Medicare studies [3, 4, 9], including a similar study of two large databases performed by Hansen et al. who reported that after adjusting for relevant patient comorbidities that UKA patients have less peri- operative complications than those having TKA [9].

Increasing UKA utilization in the Medicare population is likely multifactorial in nature and may be attributed to advances in biomaterials and implant design that reduce implant failure, increasing surgeon familiarity and expertise with the procedure, improved surgical instrumentation, and evolving surgical indications. Despite persistent debate as to superiority of fixed polyethylene bearings versus mobile polyethylene bearings or cemented versus uncemented UKA implants, overall survivability of UKA remains high and similar to that of TKA [12].

Advancements in surgical instrumentation, such as computer and robotic-assisted navigation, has also improved implant survivability [5, 19]. Expansion of surgical indications has led to reconsidering traditional UKA indications for the current Medicare patient population [12]. Despite past thinking that UKA be reserved for low weight patients, a recent meta-analysis of 6 national registries and 31 clinical studies demonstrated no increased risk for poor outcomes or revision in patients with BMI over 30 [20]. Concomitant patellofemoral joint osteoarthritis and ACL-deficient knees, traditionally contra-indications to UKA, may no longer preclude patients from undergoing UKA as Hamilton et. al found similar functional scores in UKA patients with and without patellofemoral joint degeneration [8].

High implant survivability in the context of expanding surgical indications and advanced surgical instrumentation may explain our finding that UKA utilization accelerated most greatly during 2012 to 2014 as seen in the SAF dataset. In the SAF dataset, the greatest proportion of patients receiving UKA were in the 65–69-year-old age range, followed by patients 70–74 years old. Overall implant survivability with a minimum of eight years follow-up was found to be 87.7 and 91.1% in our respective datasets, which are similar to published UKA survival rates found in cohort studies [12]. Patients over 80 years of age demonstrated almost 96% implant survivability at eight years of follow-up, which may be due to lower functional demand anticipated in this age group. Though patients’ resumption of activity was not studied in a database analysis, recent evidence suggests that 75% of UKA patients return to sport activity compared to 59% of TKA patients (*p* < 0.001) [16].

Results varied between the Humana and the SAF datasets, which demonstrates the difference in patient populations when evaluating the entire Medicare population in SAF compared to evaluating patients with Humana Medicare advantage. Humana Medicare advantage is a supplemental health insurance coverage product that may be purchased by Medicare participants that provides additional benefits to enrollees. Though the Humana dataset does not contain all Medicare patients, Humana has one of the largest number of Medicare Advantage enrollees, offering plans in 83% of all counties in the United States [11]. With socioeconomic status known as a social determinant of health, Humana Medicare Advantage participants may have higher baseline health characteristics than the average Medicare enrollee.

### Limitations

Limitations to this study additionally include its retrospective review of a large database. Demographic variables are limited to those collected by the database. The use of ICD codes to determine postoperative complications does not allow for analysis of severity. Additionally, we were unable to analyze laterality of UKA (medial versus lateral) or type of UKA bearing (fixed versus mobile). Conversion to TKA did not specify laterality, therefore in our study it was possible for a patient to have UKA and then undergo TKA on their contralateral limb. This lack of specificity overestimates conversion to TKA, and actual UKA survivability in our study may be higher than identified due to this coding limitation. Patients greater than 80 years of age experienced better implant survival rates than younger aged Medicare patients, however, this survivability may be artificially high as death may precede these patients prior to UKA implant failure and conversion to TKA.

While the PearlDiver application permits identification and analysis of patients who were prescribed opioids after UKA or TKA, the database does not link opioid prescription with CPT code. Accordingly, it is possible that patients were prescribed opioids for reasons unrelated to their UKA or TKA procedure. However, given that the mean number of days for which opioids were prescribed were 186.1 days for TKA and 144.7 days for UKA, we feel confident that the prescriptions were provided for a discrete event, such as elective surgery. One additional limitation to our opioid analysis is our limitation of using number of prescribed days as a marker of opioid use, as the database does not specify dosage or compliance, which would have been a superior method to measure opioid consumption. Opioid use analysis was not possible in the SAF dataset due to lack of data.

## Conclusion

Unicompartmental knee arthroplasty represents an increasingly utilized treatment for osteoarthritis in the Medicare population. Unicompartmental knee arthroplasty utilization may be comparatively advantageous to total knee arthroplasty due to reduced opioid use and complication rates after surgery.

## Data Availability

The datasets analyzed during the current study are available in the PearlDiver application/repository, available with subscription at http://www.pearldiverinc.com/.

## Appendix A: Elixhauser and Charlson Calculations

The average Charlson Comorbidity Index and Elixhauser Comorbidity Index were calculated for the UKA and TKA groups. These indices were calculated automatically by the PearlDiver application using the following criteria, which has been adapted from the PearlDiver Research Manual Version 2.4.

- **The Charlson Comorbidity Index** is based on the following criteria:
  - ○ Apply 1 point for each of the following:
    - ▪ Myocardial Infarction
    - ▪ Congestive Heart Failure
    - ▪ Peripheral Vascular Disease
    - ▪ Cerebrovascular Disease
    - ▪ Dementia
    - ▪ COPD
    - ▪ Connective Tissue Disease
    - ▪ Peptic Ulcer Disease
    - ▪ Diabetes Mellitus (uncomplicated)
    - ▪ Liver Disease (Mild)
  - ○ Apply 2 points for each of the following:
    - ▪ Diabetes Mellitus (end-organ damage)
    - ▪ Moderate to Severe Chronic Kidney Disease
    - ▪ Hemiplegia (2 points)
    - ▪ Leukemia (2 points)
    - ▪ Malignant Lymphoma (2 points)
    - ▪ Solid Tumor (not metastatic)
  - ○ Apply 3 points for patients with moderate to severe liver disease
  - ○ Apply 6 points for each of the following:
    - ▪ AIDS (6 points)
    - ▪ Metastatic solid tumor
  - ○ Apply the following points based on age:
    - ▪ Age <50 years: 0 points
    - ▪ Age 50-59 years: 1 points
    - ▪ Age 60-69 years: 2 points
    - ▪ Age 70-79 years: 3 points
- **The Elixhauser Comorbidity Index** is calculated based on the following criteria:
  - ○ Apply 1 point for each of the following:
    - ▪ Congestive heart failure
    - ▪ Cardiac arrhythmias
    - ▪ Valvular disease
    - ▪ Pulmonary circulation Disorders
    - ▪ Peripheral vascular disorders
    - ▪ Hypertension, uncomplicated
    - ▪ Hypertension, complicated
    - ▪ Paralysis
    - ▪ Other neurological disorders
    - ▪ Chronic pulmonary disease
    - ▪ Diabetes, uncomplicated
    - ▪ Diabetes, complicated
    - ▪ Hypothyroidism
    - ▪ Renal failure
    - ▪ Liver disease
    - ▪ Peptic ulcer disease excluding bleeding
    - ▪ AIDS/H1V
    - ▪ Lymphoma
    - ▪ Solid tumor without metastasis
    - ▪ Rheumatoid arthritis/ collagen vascular diseases
    - ▪ Coagulopathy
    - ▪ Obesity
    - ▪ Weight loss
    - ▪ Fluid and electrolyte disorders
    - ▪ Blood loss anemia
    - ▪ Deficiency anemia
    - ▪ Alcohol abuse
    - ▪ Drug abuse
    - ▪ Psychoses
    - ▪ Depression

## Appendix B: Opioids

Opioid use was defined as patients filing prescriptions for any of the following drugs. This list was provided by PearlDiver staff as acknowledged.

- HYDROCODONE BITARTRATE AND ACETAMINOPHEN
- MORPHINE SULFATE
- FENTANYL TRANSDERMAL SYSTEM
- METHADONE HYDROCHLORIDE
- CODEINE PHOSPHATE, PHENYLEPHRINE HYDROCHLORIDE
- HYDROCODONE BITARTRATE,
- OXYCODONE HYDROCHLORIDE AND ACETAMINOPHEN
- OXYCODONE HYDROCHLORIDE
- FENTANYL
- DIHYDROCODEINE BITARTRATE, BROMPHENIRAMINE MALEATE, PSEUDOEPHEDRINE HYDROCHLORIDE
- BUPRENORPHINE HYDROCHLORIDE
- HYDROMORPHONE HYDROCHLORIDE
- HYDROMORPHONE HCL
- FENTANYL CITRATE
- OXYCODONE
- OXYCODONE AND ACETAMINOPHEN
- BUPRENORPHINE HYDROCHLORIDE AND NALOXONE HYDROCHLORIDE DIHYDRATE
- OXYMORPHONE HYDROCHLORIDE
- CODEINE PHOSPHATE, GUAIFENESIN AND PSEUDOEPHEDRINE HYDROCHLORIDE
- HYDROCODONE BITARTRATE AND IBUPROFEN
- MEPERIDINE HCL
- TAPENTADOL
- MEPERIDINE HYDROCHLORIDE
- HYDROCODONE BITARTRATE, ACETAMINOPHEN
- HYDROCODONE POLISTIREX AND CHLORPHENIRAMINE POLISTIREX
- TAPENTADOL HYDROCHLORIDE
- HYDROCODONE BITARTRATE
- CODEINE PHOSPHATE AND GUAIFENESIN
- NORETHINDRONE AND ETHINYL ESTRADIOL TABLETS
- BUPRENORPHINE AND NALOXONE
- BUPRENORPHINE HYDROCHLORIDE AND NALOXONE HYDROCHLORIDE
- CODEINE PHOSPHATE, DEXCHORPHENIRAMINE MALEATE, PHENYLEPHRINE HYDROCHLORIDE
- FENTANYL CITRATE, BUPIVACAINE HCL
- MORPHINE SULFATE AND NALTREXONE HYDROCHLORIDE
- BUPRENORPHINE HYDROCHLORIDE, NALOXONE HYDROCHLORIDE
- HYDROCODONE BITARTRATE, IBUPROFEN
- CODEINE PHOSPHATE, PROMETHAZINE HYDROCHLORIDE, AND PHENYLEPHRINE HYDROCHLORIDE
- MORPHINE
- BUPRENORPHINE HCL
- FENTANYL BUCCAL
- CODEINE PHOSPHATE AND APAP
- CODEINE PHOSPHATE, GUAIFENESIN, PSEUDOEPHEDRINE HYDROCHLORIDE
- MORPHINE SUFATE
- CODEINE PHOSPHATE, PHENYLEPHRINE HYDROCHLORIDE, CHLORPHENIRAMINE MALEATE
- BUPRENORPHINE HYDROCHLORIDE SUBLINGUAL
- HYDROCODONE BITARTRATE AND CHLORPHENIRAMINE MALEATE
- CODEINE SULFATE
- HYDROCODONE BITATRATE AND ACETAMINOPHEN
- HYDROCODONE BITARTRATE, AND CHLORPHENIRAMINE MALEATE
- HYDROCODONE BITARTRATE, CHLORPHENIRAMINE MALEATE AND PSEUDOEPHEDRINE HYDROCHLORIDE
- MORPHINE BASE
- BUPRENORPHINE
- HYDROCODONE BITARTATE AND ACETAMINOPHEN
- OXYCODONE HYDROCHLORIDE, ACETAMINOPHEN
- HYDROCODONE BITARTRATE, CHLORPHENIRAMINE MALEATE, AND PSEUDOEPHEDRINE HYDROCHLORIDE
- HYDROCODONE POLISTIREX AND CHLORPHENIRAMINE POLISITREX
- OXYCODONE AND ASPIRIN
- DIHYDROCODEINE BITARTRATE, PHENYLEPHRINE HYDROCHLORIDE, PYRILAMINE MALEATE
- CODEINE PHOSPHATE, PHENYLEPHRINE HYDROCHLORIDE, BROMPHENIRAMINE MALEATE
- OXYCODONE HCL CONTROLLED-RELEASE
- CODEINE PHOSPHATE
- CODEINE PHOSPHATE, DEXBROMPHENIRAMINE MALEATE, PSEUDOEPHEDRINE HYDROCHLORIDE
- CODEINE PHOSPHATE AND GUAIFENESIN AND PSEUDOPHEDRINE
- HYDROCODONE BITARTRATE, CHLORPHENIRAMINE MALEATE, PSEUDOEPHEDRINE HYDROCHLORIDE
- MORPHINE HYDROCHLORIDE
- HYDROCODONE, ACETAMINOPHEN
- MORPHINE SULFATE EXTENDED RELEASE
- HYDROCODONE BITARTRATE, ACETAMINOPHEN, .GAMMA.-AMINOBUTYRIC ACID
- CODEINE PHOSPHATE, PHENYLEPHRINE HYDROCHLORIDE AND PYRILAMINE MALEATE
- CODEINE PHOSPHATE, PSEUDOEPHEDRINE HYDROCHLORIDE, BROMPHENIRAMINE MALEATE
- CODEINE PHOSPHATE, PSEUDOEPHEDRINE HYDROCHLORIDE, PYRILAMINE MALEATE
- OXYCODONE AND ASPIRIN TABLETS
- CODEINE PHOSPHATE AND CHLORPHENIRAMINE MALEATE
- DIHYDROCODEINE BITARTRATE, PHENYLEPHRINE HYDROCHLORIDE, AND GUAIFENESIN
- CODEINE PHOSPHATE, GUAIFENESIN
- DIHYDROCODEINE BITARTRATE, GUAIFENESIN
- OXYCODONE HYDROCHLORIDE AND IBUPROFEN
- CODEINE PHOSPHATE, GUAIFENESIN, AND PSEUDOEPHEDRINE HYDROCHLORIDE
- HYDROCODONE BITARTRATE AND PSEUDOEPHEDRINE HYDROCHLORIDE
- CODEINE PHOSPHATE, PSEUDOEPHEDRINE HCL, CHLORCYCLIZINE HCL
- OXYCODONE HYDCHLORIDE
- DIHYDROCODEINE BITARTRATE, BROMPHENIRAMINE MALEATE, AND PHENYLEPHRINE HYDROCHLORIDE
- HYDROCODONE BITARTRATE AND ACETAMINPHEN
- MORPHINE SULFATE ORAL SOLUTION
- OXYCODONE HYDROCHLORIDE AND ASPIRIN
- CODEINE PHOSPHATE AND PYRILAMINE MALEATE
- HYDROCODONE POLISTIREX AND CHLORPHENIRAMINE POLISTIREX EXTENDED RELEASE ORAL SUSPENSION
- DIHYDROCODEINE BITARTRATE, PHENYLEPHRINE HYDROCHLORIDE, GUAIFENESIN
- CODEINE PHOSPHATE, BUTALBITAL, CAFFEINE, AND ACETAMINOPHEN CAPSULE
- DIHYDROCODEINE BITARTRATE, ACETAMINOPHEN AND CAFFEINE
- HYDROCODONE BITARTRATE WITH ACETAMINOPHEN
- CODEINE PHOSPHATE, PHENYLEPHRINE HYDROCHLORIDE AND DIPHENHYDRAMINE HYDROCHLORIDE
- OXYCODONE HCL AND ACETAMINOPHEN
- CODEINE BASE
- HYDROCODONE BITARTRATE, PSEUDOEPHEDRINE HYDROCHLORIDE
- CODEINE PHOSPHATE, PSEUDOEPHEDRINE HYDROCHLORIDE
- HYDROCODONE, ACETAMINOPHEN, .GAMMA.-AMINOBUTYRIC ACID
- MEPERIDINE HYDROCHLORIDE AND PROMETHAZINE HYDROCHLORIDE
- CODEINE PHOSPHATE, CHLORCYCLIZINE HCL
- HYDROCODONE BITARTATE AND HOMATROPINE METHYLBROMIDE
- CODEINE PHOSPHATE, GUIAFENESIN, PSEUDOEPHEDRINE HCL
- CODEINE PHOSPHATE / GUAIFENESIN
- HYDROCODONE BITARTRATE AND GUAIFENESIN
- CODEINE PHOSPHATE/GUAIFENESIN/PSEUDOEPHEDRINE HYDROCHLORIDE
- MORPHINE TINCTURE

## Appendix C: Complications

Complications were defined by their respective ICD-9 and ICD-10 codes in cohorts predefined by the PearlDiver application. The codes used for each complication are listed below, adapted from the PearlDiver Research Manual Version 2.4.

- **Acute Kidney Injury**: ICD-9-D-5845,ICD-9-D-5846,ICD-9-D-5847,ICD-9-D-5848,ICD-9-D-5849,ICD-10-D-N17:ICD-10-D-N179
- **Cardiac Arrest**: ICD-9-D-4275,ICD-9-D-42741,ICD-10-D-I46:ICD-10-D-I469
- **Deep Vein Thrombosis (DVT):** ICD-9-D-4532,ICD-9-D-4533,ICD-9-D-4534,ICD-9-D-45382,ICD-9-D-45384,ICD-9-D-45385,ICD-9-D-45386,ICD-10-D-I26:ICD-10-D-I2699
- **Nerve Injury**: ICD-9-D-9550,ICD-9-D-9551,ICD-9-D-9552,ICD-9-D-9553,ICD-9-D-9554,ICD-9-D-9555,ICD-9-D-9556,ICD-9-D-9557,ICD-9-D-9558,ICD-9-D-9559,ICD-9-D-9074,ICD-10-D-S440,ICD-10-DS4400,ICD-10-D-S4400XA,ICD-10-D-S4400XD,ICD-10-D-S4400XS,ICD-10-D-S4401,ICD-10-DS4401XA,ICD-10-D-S4401XD,ICD-10-D-S4401XS,ICD-10-D-S4402,ICD-10-D-S4402XA,ICD-10-DS4402XD,ICD-10-D-S4402XS,ICD-10-D-S441,ICD-10-D-S4410,ICD-10-D-S4410XA,ICD-10-D-S4410XD,ICD-10-D-S4410XS,ICD-10-D-S4411,ICD-10-D-S4411XA,ICD-10-D-S4411XD,ICD-10-D-S4411XS,ICD-10-DS4412,ICD-10-D-S4412XA,ICD-10-D-S4412XD,ICD-10-D-S4412XS,ICD-10-D-S442,ICD-10-D-S4420,ICD-10-D-S4420XA,ICD-10-D-S4420XD,ICD-10-D-S4420XS,ICD-10-D-S4421,ICD-10-D-S4421XA,ICD-10-DS4421XD,ICD-10-D-S4421XS,ICD-10-D-S4422,ICD-10-D-S4422XA,ICD-10-D-S4422XD,ICD-10-DS4422XS,ICD-10-D-S443,ICD-10-D-S4430,ICD-10-D-S4430XA,ICD-10-D-S4430XD,ICD-10-D-S4430XS,ICD-10-D-S4431,ICD-10-D-S4431XA,ICD-10-D-S4431XD,ICD-10-D-S4431XS,ICD-10-D-S4432,ICD-10-DS4432XA,ICD-10-D-S4432XD,ICD-10-D-S4432XS,ICD-10-D-S444,ICD-10-D-S4440,ICD-10-D-S4440XA,ICD-10-D-S4440XD,ICD-10-D-S4440XS,ICD-10-D-S4441,ICD-10-D-S4441XA,ICD-10-D-S4441XD,ICD-10-DS4441XS,ICD-10-D-S4442,ICD-10-D-S4442XA,ICD-10-D-S4442XD,ICD-10-D-S4442XS,ICD-10-D-S445,ICD-10-D-S4450,ICD-10-D-S4450XA,ICD-10-D-S4450XD,ICD-10-D-S4450XS,ICD-10-D-S4451,ICD-10-DS4451XA,ICD-10-D-S4451XD,ICD-10-D-S4451XS,ICD-10-D-S4452,ICD-10-D-S4452XA,ICD-10-DS4452XD,ICD-10-D-S4452XS,ICD-10-D-S448,ICD-10-D-S448X,ICD-10-D-S448X1,ICD-10-D-S448X1A,ICD-10-D-S448X1D,ICD-10-D-S448X1S,ICD-10-D-S448X2,ICD-10-D-S448X2A,ICD-10-D-S448X2D,ICD-10-DS448X2S,ICD-10-D-S448X9,ICD-10-D-S448X9A,ICD-10-D-S448X9D,ICD-10-D-S448X9S,ICD-10-DS449,ICD-10-D-S4490,ICD-10-D-S4490XA,ICD-10-D-S4490XD,ICD-10-D-S4490XS,ICD-10-D-S4491, ICD-10-D-S4491XA,ICD-10-D-S4491XD,ICD-10-D-S4491XS,ICD-10-D-S4492,ICD-10-D-S4492XA,ICD-10-DS4492XD,ICD-10-D-S4492XS
- **Pneumonia:** ICD-9-D-4800:ICD-9-D-4809,ICD-9-D-481,ICD-9-D-4820,ICD-9-D-4821,ICD-9-D-48230,ICD-9-D-48231,ICD-9-D-48232,ICD-9-D-48239,ICD-9-D-48240,ICD-9-D-48241,ICD-9-D-48242,ICD-9-D-48249,ICD-9-D-48281,ICD-9-D-48282,ICD-9-D-48283,ICD-9-D-48284,ICD-9-D-48289,ICD-9-D-4829,ICD-9-D-4830,ICD-9-D-4831,ICD-9-D-4838,ICD-9-D-4841,ICD-9-D-4843,ICD-9-D-4845,ICD-9-D-4846,ICD-9-D-4847,ICD-9-D-4848,ICD-9-D-485,ICD-9-D-486,ICD-10-D-J12:ICD-10-D-J189
- **Pulmonary Embolism:** ICD-9-D-4151:ICD-9-D-4159,ICD-10-D-I26:ICD-10-D-I269
- **Urinary Tract Infection:** ICD-9-D-5990,ICD-10-D-N390
- **Disruption of Wound:** ICD-9-D-99830,ICD-9-D-99831,ICD-9-D-99832,ICD-9-D-99833,ICD-10-DT8130XA,ICD-10-D-T8130XD,ICD-10-D-T8130XS,ICD-10-D-T8131XA,ICD-10-D-T8131XD,ICD-10-DT8131XS,ICD-10-D-T8132XA,ICD-10-D-T8132XD,ICD-10-D-T8132XS,ICD-10-D-T8133XA,ICD-10-DT8133XD,ICD-10-D-T8133XS
- **Hematoma:** ICD-9-D-99811,ICD-9-D-99812,ICD-9-D-99813,ICD-10-D-D7801, ICD-10-D-D7802,ICD-10-D-D7821, ICD-10-D-D7822, ICD-10-D-E3601, ICD-10-D-E3602, ICD-10-D-E89810, ICD-10-D-E89811,ICD-10-D-G9731, ICD-10-D-G9732, ICD-10-D-G9751, ICD-10-D-G9752, ICD-10-D-H59111, ICD-10-DH59112,ICD-10-D-H59113, ICD-10-D-H59119, ICD-10-D-H59121, ICD-10-D-H59122, ICD-10-D-H59123,ICD-10-D-H59129, ICD-10-D-H59311, ICD-10-D-H59312, ICD-10-D-H59313, ICD-10-D-H59319, ICD-10-DH59321,ICD-10-D-H59322, ICD-10-D-H59323, ICD-10-D-H59329, ICD-10-D-H9521, ICD-10-D-H9522, ICD-10-D-H9541, ICD-10-D-H9542, ICD-10-D-I97410, ICD-10-D-I97411, ICD-10-D-I97418, ICD-10-D-I9742, ICD-10-D-I97610, ICD-10-D-I97611, ICD-10-D-I97618, ICD-10-D-I97620, ICD-10-D-J9561, ICD-10-D-J9562, ICD-10-D-J95830, ICD-10-D-J95831, ICD-10-D-K9161, ICD-10-D-K9162, ICD-10-D-K91840, ICD-10-D-K91841,ICD-10-D-L7601, ICD-10-D-L7602, ICD-10-D-L7621, ICD-10-D-L7622, ICD-10-D-M96810, ICD-10-DM96811,ICD-10-D-M96830, ICD-10-D-M96831, ICD-10-D-N9961, ICD-10-D-N9962, ICD-10-D-N99820, ICD-10-D-N99821, ICD-10-D-T888XXA
- **Transfusion:** ICD-9-P-9904,ICD-10-P-3023,ICD-10-P-30230AZ,ICD-10-P-30230G0,ICD-10-P-30230G2,ICD-10-P-30230G3,ICD-10-P-30230G4,ICD-10-P-30230H0,ICD-10-P-30230H1,ICD-10-P-30230J0,ICD-10-P-30230J1,ICD-10-P-30230K0,ICD-10-P-30230K1,ICD-10-P-30230L0,ICD-10-P-30230L1,ICD-10-P-30230M0,ICD-10-P-30230M1,ICD-10-P-30230N0,ICD-10-P-30230N1,ICD-10-P-30230P0,ICD-10-P-30230P1,ICD-10-P-30230Q0,ICD-10-P-30230Q1,ICD-10-P-30230R0,ICD-10-P-30230R1,ICD-10-P-30230S0,ICD-10-P-30230S1,ICD-10-P-30230T0,ICD-10-P-30230T1,ICD-10-P-30230V0,ICD-10-P-30230V1,ICD-10-P-30230W0,ICD-10-P-30230W1,ICD-10-P-30230X0,ICD-10-P-30230X2,ICD-10-P-30230X3,ICD-10-P-30230X4,ICD-10-P-30230Y0,ICD-10-P-30230Y2,ICD-10-P-30230Y3,ICD-10-P-30230Y4,ICD-10-P-30233AZ,ICD-10-P-30233G0,ICD-10-P-30233G2,ICD-10-P-30233G3,ICD-10-P-30233G4,ICD-10-P-30233H0,ICD-10-P-30233H1,ICD-10-P-30233J0,ICD-10-P-30233J1,ICD-10-P-30233K0,ICD-10-P-30233K1,ICD-10-P-30233L0,ICD-10-P-30233L1,ICD-10-P-30233M0,ICD-10-P-30233M1,ICD-10-P-30233N0,ICD-10-P-30233N1,ICD-10-P-30233P0,ICD-10-P-30233P1,ICD-10-P-30233Q0,ICD-10-P-30233Q1,ICD-10-P-30233R0,ICD-10-P-30233R1,ICD-10-P-30233S0,ICD-10-P-30233S1,ICD-10-P-30233T0,ICD-10-P-30233T1,ICD-10-P-30233V0,ICD-10-P-30233V1,ICD-10-P-30233W0,ICD-10-P-30233W1,ICD-10-P-30233X0,ICD-10-P-30233X2,ICD-10-P-30233X3,ICD-10-P-30233X4,ICD-10-P-30233Y0,ICD-10-P-30233Y2,ICD-10-P-30233Y3,ICD-10-P-30233Y4,ICD-10-P-30240AZ,ICD-10-P-30240G0,ICD-10-P-30240G2,ICD-10-P-30240G3,ICD-10-P-30240G4,ICD-10-P-30240H0,ICD-10-P-30240H1,ICD-10-P-30240J0,ICD-10-P-30240J1,ICD-10-P-30240K0,ICD-10-P-30240K1,ICD-10-P-30240L0,ICD-10-P-30240L1,ICD-10-P-30240M0,ICD-10-P-30240M1,ICD-10-P-30240N0,ICD-10-P-30240N1,ICD-10-P-30240P0,ICD-10-P-30240P1,ICD-10-P-30240Q0,ICD-10-P-30240Q1,ICD-10-P-30240R0,ICD-10-P-30240R1,ICD-10-P-30240S0,ICD-10-P-30240S1,ICD-10-P-30240T0,ICD-10-P-30240T1,ICD-10-P-30240V0,ICD-10-P-30240V1,ICD-10-P-30240W0,ICD-10-P-30240W1,ICD-10-P-30240X0,ICD-10-P-30240X2,ICD-10-P-30240X3,ICD-10-P-30240X4,ICD-10-P-30240Y0,ICD-10-P-30240Y2,ICD-10-P-30240Y3,ICD-10-P-30240Y4,ICD-10-P-30243AZ,ICD-10-P-30243G0,ICD-10-P-30243G2,ICD-10-P-30243G3,ICD-10-P-30243G4,ICD-10-P-30243H0,ICD-10-P-30243H1,ICD-10-P-30243J0,ICD-10-P-30243J1,ICD-10-P-30243K0,ICD-10-P-30243K1,ICD-10-P-30243L0,ICD-10-P-30243L1,ICD-10-P-30243M0,ICD-10-P-30243M1,ICD-10-P-30243N0,ICD-10-P-30243N1,ICD-10-P-30243P0,ICD-10-P-30243P1,ICD-10-P-30243Q0,ICD-10-P-30243Q1,ICD-10-P-30243R0,ICD-10-P-30243R1,ICD-10-P-30243S0,ICD-10-P-30243S1,ICD-10-P-30243T0,ICD-10-P-30243T1,ICD-10-P-30243V0,ICD-10-P-30243V1,ICD-10-P-30243W0,ICD-10-P-30243W1,ICD-10-P-30243X0,ICD-10-P-30243X2,ICD-10-P-30243X3,ICD-10-P-30243X4,ICD-10-P-30243Y0,ICD-10-P-30243Y2,ICD-10-P-30243Y3,ICD-10-P-30243Y4,ICD-10-P-30250G0,ICD-10-P-30250G1,ICD-10-P-30250H0,ICD-10-P-30250H1,ICD-10-P-30250J0,ICD-10-P-30250J1,ICD-10-P-30250K0,ICD-10-P-30250K1,ICD-10-P-30250L0,ICD-10-P-30250L1,ICD-10-P-30250M0,ICD-10-P-30250M1,ICD-10-P-30250N0,ICD-10-P-30250N1,ICD-10-P-30250P0,ICD-10-P-30250P1,ICD-10-P-30250Q0,ICD-10-P-30250Q1,ICD-10-P-30250R0,ICD-10-P-30250R1,ICD-10-P-30250S0,ICD-10-P-30250S1,ICD-10-P-30250T0,ICD-10-P-30250T1,ICD-10-P-30250V0,ICD-10-P-30250V1,ICD-10-P-30250W0,ICD-10-P-30250W1,ICD-10-P-30250X0,ICD-10-P-30250X1,ICD-10-P-30250Y0,ICD-10-P-30250Y1,ICD-10-P-30253G0,ICD-10-P-30253G1,ICD-10-P-30253H0,ICD-10-P-30253H1,ICD-10-P-30253J0,ICD-10-P-30253J1,ICD-10-P-30253K0,ICD-10-P-30253K1,ICD-10-P-30253L0,ICD-10-P-30253L1,ICD-10-P-30253M0,ICD-10-P-30253M1,ICD-10-P-30253N0,ICD-10-P-30253N1,ICD-10-P-30253P0,ICD-10-P-30253P1,ICD-10-P-30253Q0,ICD-10-P-30253Q1,ICD-10-P-30253R0,ICD-10-P-30253R1,ICD-10-P-30253S0,ICD-10-P-30253S1,ICD-10-P-30253T0,ICD-10-P-30253T1,ICD-10-P-30253V0,ICD-10-P-30253V1,ICD-10-P-30253W0,ICD-10-P-30253W1,ICD-10-P-30253X0,ICD-10-P-30253X1,ICD-10-P-30253Y0,ICD-10-P-30253Y1,ICD-10-P-30260G0,ICD-10-P-30260G1,ICD-10-P-30260H0,ICD-10-P-30260H1,ICD-10-P-30260J0,ICD-10-P-30260J1,ICD-10-P-30260K0,ICD-10-P-30260K1,ICD-10-P-30260L0,ICD-10-P-30260L1,ICD-10-P-30260M0,ICD-10-P-30260M1,ICD-10-P-30260N0,ICD-10-P-30260N1,ICD-10-P-30260P0,ICD-10-P-30260P1,ICD-10-P-30260Q0,ICD-10-P-30260Q1,ICD-10-P-30260R0,ICD-10-P-30260R1,ICD-10-P-30260S0,ICD-10-P-30260S1,ICD-10-P-30260T0,ICD-10-P-30260T1,ICD-10-P-30260V0,ICD-10-P-30260V1,ICD-10-P-30260W0,ICD-10-P-30260W1,ICD-10-P-30260X0,ICD-10-P-30260X1,ICD-10-P-30260Y0,ICD-10-P-30260Y1,ICD-10-P-30263G0,ICD-10-P-30263G1,ICD-10-P-30263H0,ICD-10-P-30263H1,ICD-10-P-30263J0,ICD-10-P-30263J1,ICD-10-P-30263K0,ICD-10-P-30263K1,ICD-10-P-30263L0,ICD-10-P-30263L1,ICD-10-P-30263M0,ICD-10-P-30263M1,ICD-10-P-30263N0,ICD-10-P-30263N1,ICD-10-P-30263P0,ICD-10-P-30263P1,ICD-10-P-30263Q0,ICD-10-P-30263Q1,ICD-10-P-30263R0,ICD-10-P-30263R1,ICD-10-P-30263S0,ICD-10-P-30263S1,ICD-10-P-30263T0,ICD-10-P-30263T1,ICD-10-P-30263V0,ICD-10-P-30263V1,ICD-10-P-30263W0,ICD-10-P-30263W1,ICD-10-P-30263X0,ICD-10-P-30263X1, ICD-10-P-30263Y0,ICD-10-P-30263Y1,ICD-10-P-30273H1,ICD-10-P-30273J1,ICD-10-P-30273K1,ICD-10-P-30273L1,ICD-10-P-30273M1,ICD-10-P-30273N1,ICD-10-P-30273P1,ICD-10-P-30273Q1,ICD-10-P-30273R1,ICD-10-P-30273S1,ICD-10-P-30273T1,ICD-10-P-30273V1,ICD-10-P-30273W1,ICD-10-P-30277H1,ICD-10-P-30277J1,ICD-10-P-30277K1,ICD-10-P-30277L1,ICD-10-P-30277M1,ICD-10-P-30277N1,ICD-10-P-30277P1,ICD-10-P-30277Q1,ICD-10-P-30277R1,ICD-10-P-30277S1,ICD-10-P-30277T1,ICD-10-P-30277V1,ICD-10-P-30277W1,ICD-10-P-30280B1,ICD-10-P-30283B1
- **Capsulitis:** ICD-9-D-7260,ICD-9-D-71951,ICD-10-D-M7500,ICD-10-D-M7501,ICD-10-D-M7502

## Abbreviations

CPT: Current Procedural Terminology
DVT: Deep vein thrombosis
HIPAA: Health Insurance Portability and Accountability Act
ICD-9: International Classification of Diseases, Ninth Revision
ICD-10: International Classification of Diseases, Tenth Revision
OA: Osteoarthritis
SAF: Medicare Standard Analytic File
SD: Standard deviation
TKA: Total knee arthroplasty
UKA: Unicompartmental knee arthroplasty
